# Plant responses to cold stress: molecular and physiological adaptive mechanisms with breeding advances

**DOI:** 10.3389/fpls.2026.1810324

**Published:** 2026-05-29

**Authors:** Haixia Li, Lin Cui, Song Liu, Tianhe Zhang, Yuexia Wang, Xiaoxing Wang, Haifang Jiang

**Affiliations:** 1 State Key Laboratory of Wheat & Maize Crop Science/College of Life Sciences, Henan Agricultural University, Zhengzhou, China; 2 Maize Research Center, Hebi Academy of Agricultural Sciences, Hebi, China

**Keywords:** cold stress, crop, molecular breeding, molecular mechanism, regulatory networks

## Abstract

Cold stress is a critical bottleneck that constrains crop cultivation boundaries, reshapes species distribution patterns, and limits yield improvement, directly threatening plant survival and growth. This review systematically analyzes the impacts of cold stress on plants with cold stress as its core research focus. Different from superficial descriptive studies, this review conducts an in-depth analysis of the molecular mechanisms underlying plant cold tolerance, systematically dissects the complete molecular pathway involving the synergistic interaction among cold signal perception, transduction and downstream response components, comprehensively presents cutting-edge research advances in this field, and clarifies the previously abstract molecular mechanisms. By screening key candidate genes responsive to cold stress, this study deciphers the molecular regulatory code underlying crop cold tolerance and characterizes the corresponding regulatory network, which provides valuable genetic resources and solid theoretical support for molecular breeding of cold-tolerant crops and specifies the direction for the cultivation of cold-tolerant high-yield crops. This research closely responds to national food security demands and bridges laboratory-based molecular exploration with practical field applications. The research outcomes can enhance the stress-resilience of agricultural production, and are of great strategic significance for consolidating the national food security barrier and promoting the high-quality development of agriculture.

## 1 Introduction

In natural ecosystems, plant growth is closely intertwined with external environmental factors. To enhance survival within complex and dynamic surroundings, plants continually adjust their physiological states and metabolic processes in response to environmental fluctuations. Unlike animals, plants are sessile organisms that cannot rapidly evade sudden environmental stresses. Throughout long-term evolution, plants have developed distinctive and efficient adaptive mechanisms to cope with diverse environmental stressors. These stressors are broadly categorized into biotic and abiotic types (Ding et al., 2018). Biotic stress results from damage inflicted by living organisms, such as insects, pathogens, and harmful microorganisms, which may impair normal plant growth and development through herbivory, parasitism, or infection. Abiotic stress, on the other hand, arises from adverse physical conditions including low or high temperature, drought, flooding, salinity, light pollution, radiation, and mechanical injury. These factors can disrupt key physiological processes like photosynthesis, respiration, and water metabolism, ultimately leading to growth inhibition, yield reduction, or even plant mortality (Guan et al., 2023; Laxman et al., 2022; Lv et al., 2021; Zhu, 2016).

To adapt to cold stress, plants have evolved complex and precise regulatory mechanisms over long-term evolution. Among these, cold acclimation (CA) represents a key adaptive strategy (Thomashow, 1999). During CA, plants undergo a range of physiological and biochemical adjustments, including changes in cell membrane fluidity, accumulation of osmoregulatory compounds, enhanced antioxidant enzyme activities, dynamic changes of various protective metabolites, and regulation of gene expression. For the majority of thermophilic plants, low temperatures reduce membrane fluidity, thereby impairing the uptake and translocation of water and mineral nutrients. These modifications collectively enhance plant cold tolerance, facilitating better survival under cold conditions (Xu et al., 2020; Zhang et al., 2025).

## 2 Effects of cold stress on plant growth, development, and physiological regulation

When subjected to short-term chilling stress, plants typically exhibit acute physiological symptoms, including rapid leaf wilting and tissue softening. These phenotypic alterations are closely linked to decreased cell membrane fluidity, disruption of ion homeostasis, and impaired water transport (Ding et al., 2015; Feng et al., 2025). The effects of long-term chilling stress on plant growth and development display stage-specific patterns. During vegetative growth, chilling impairs shoot apical meristem activity, reduces leaf expansion rates, and compromises root absorption capacity. In reproductive stages, cold conditions frequently cause pollen abortion, aberrant ovule development, and disruption of pollination processes, ultimately leading to floral sterility (Ding and Yang, 2022). Such physiological disturbances often result in reduced seed-setting rates and diminished fruit quality in agricultural systems, thereby posing risks to global food security. Furthermore, chilling stress induce biofilm solidification, which impedes water uptake by plants and results in physiological drought.

As central sites of photosynthesis, chloroplasts exhibit high sensitivity to cold stress. Under chilling conditions, increased thylakoid membrane viscosity restricts plastoquinone (PQ) diffusion and impairs photosynthetic electron transport, leading to functional disruption of photosystem I (PSI) and photosystem II (PSII), and ultimately inducing photooxidative damage (Griffith et al., 1984; Suzuki et al., 2012). While nitrogen enrichment can enhance cold tolerance in maize to some degree, while also increasing chlorophyll and Rubisco content and improving PSII efficiency (Soualihou et al., 2023).

**Figure 1:**
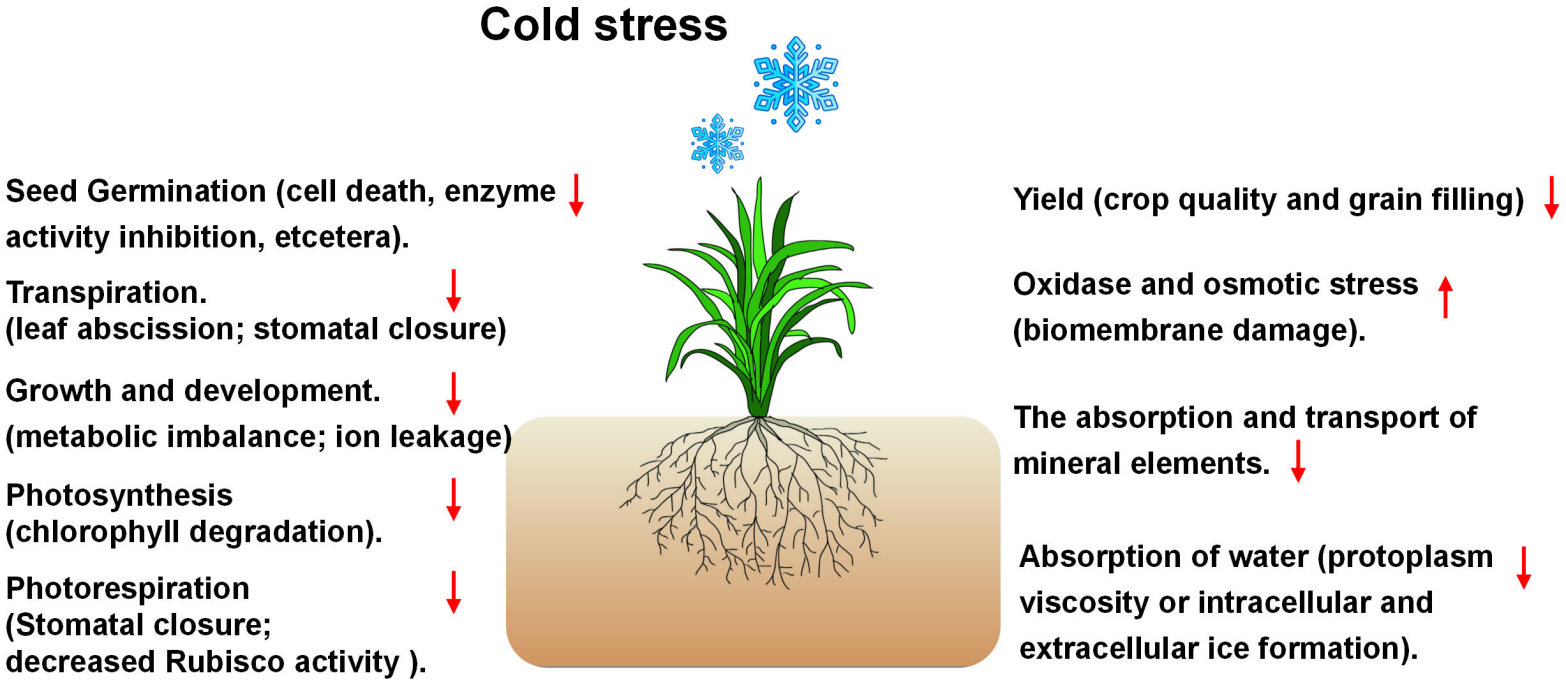
Effects of cold stress on plants. Cold stress profoundly suppresses key physiological and biochemical processes, including seed germination, transpiration, vegetative and reproductive growth, photosynthesis, photorespiration, mineral nutrient uptake and translocation, and root water absorption, thereby compromising crop yield and quality. Concurrently, it triggers oxidative and osmotic stress, resulting in lipid peroxidation, loss of membrane integrity, and cellular dysfunction. Collectively, these perturbations impose multilayered constraints on plant growth, development, and agronomic performance. An upward arrow indicates that this physiological process is up-regulated under cold stress, while a downward arrow indicates that this physiological process is down-regulated.

The redox state alteration represents another key signaling mechanism in plant cold responses. Chilling stress induces excessive reactive oxygen species (ROS) accumulation in chloroplasts and mitochondria, leading to oxidative stress that triggers membrane lipid peroxidation and protein carbonylation. To counteract such oxidative damage, plants activate antioxidant defense systems involving superoxide dismutase (SOD), catalase (CAT), and ascorbate peroxidase (APX) (Ding et al., 2019; Ding et al., 2020; Ramazan et al., 2021; Ritonga and Chen, 2020; Suzuki et al., 2012). Additionally, cold stress cause depolymerization of cytoskeletal microtubules and microfilaments, disrupting cell morphology maintenance and intracellular transport. Concurrently, alterations in RNA secondary structure stability may contribute to cold signal transduction by modulating mRNA translation efficiency (Miura and Furumoto, 2013). The effects of cold stress on plant growth, development, and physiological metabolic processes are illustrated in the following figure (**Figure 1**).

## 3 Molecular and physiological mechanisms of plants in response to cold stress

Cold stress effectively induce the synthesis of antifreeze proteins (AFPs). These plant-derived antifreeze proteins exhibit high recrystallization inhibition activity, thereby effectively suppressing the formation of large ice crystals within tissues (Zhang et al., 2024). Moreover, the cold-induced antifreeze proteins possess dual molecular functions, combining hydrolytic activity with antifreeze properties (Zhu, 2016). In overwintering plants, the production of antifreeze proteins is commonly linked to ethylene signaling, dehydration, and short-day conditions (Griffith and Yaish, 2004). However, a sharp temperature increase following freezing injury may cause rapid melting of intercellular ice crystals. The resulting water cannot be absorbed by the cells promptly and is rapidly lost via transpiration, leading to irreversible cellular dehydration and ultimately causing plants to wilt and die (Li et al., 2022a; Wu et al., 2023). Such abrupt temperature shifts often induce secondary damage more severe than the initial freezing injury, highlighting the need for coordinated regulation between freezing tolerance and thawing adaptation in plants.

Proline and soluble sugars, as the principal osmotic adjustment substances, accumulate in response to cold-induced reprogramming of carbohydrate metabolism. Proline confers protection via a dual mechanism: its zwitterionic structure interacts with polar groups on protein surfaces, enhancing protein solubility and suppressing aggregation (Liu et al., 2007), concurrently, proline forms a hydrophilic colloidal network within cells that binds free water via hydrogen bonds, thereby lowering the freezing point of intracellular fluid and mitigating water loss (Lalk and Dorffling, 1985). Studies have indicated that Abscisic Acid (ABA) can modulate proline homeostasis under cold stress by suppressing H_2_O_2_ generation (Li et al., 2022b). Cold stress also upregulates the expression of key enzymes involved in starch degradation, such as β-amylase and debranching enzyme, which promoting the hydrolysis of starch into glucose, fructose, and sucrose (Kaplan et al., 2007). These soluble sugars lower cellular osmotic potential, reduce water efflux, and form a hydration layer on the membrane surface that prevents direct contact between ice crystals and lipid bilayers. Furthermore, soluble sugars function as molecular chaperones, helping to maintain protein conformation and suppress the loss of enzymatic activity under cold conditions (Strand et al., 2000). Root irrigation using glucose significantly increases the soluble sugar content, including glucose, fructose, and sucrose, in melons, thereby improving their cold resistance (Li et al., 2024a). Furthermore, the accumulation of anthocyanins under cold stress enhances plant cold tolerance to some degree (Bi et al., 2017; Zhang et al., 2020).

Under normal conditions, ROS in plants are maintained in a state of dynamic equilibrium. However, when subjected to stress, this ROS homeostasis is disrupted, often leading to excessive ROS accumulation (Castro et al., 2021). RmZAT10 enhances cold tolerance in roses by regulating proline and ROS homeostasis (Luo et al., 2022). Cold stress markedly upregulates the expression of *OsPOX1*. The protein encoded by this gene catalyzes the decomposition of H_2_O_2_ into water and oxygen, thereby efficiently eliminating excess ROS. Suppression of *OsPOX1* expression via RNAi leads to increased accumulation of malondialdehyde (MDA) and H_2_O_2_ in rice seedlings under cold conditions, accompanied by more pronounced leaf yellowing and growth retardation. In contrast, *OsPOX1*-overexpressing lines exhibit enhanced ROS-scavenging capacity and greater membrane stability, along with significantly improved cold tolerance (Rezaie et al., 2020). Overexpression of *NCED5* and *NCED4* in rice significantly improves cold tolerance. *OsNCED5* and *OsNCED4* encodes an ABA biosynthesis enzyme localized in chloroplasts, which primarily enhances cold tolerance by regulating ROS homeostasis (Xiang et al., 2024, Xiang et al., 2025b). Heterologous expression of the sucrose synthase VaSUS2 in tomatoes not only promotes the accumulation of glucose and fructose in tomatoes, but exhibits higher antioxidant enzyme activities and the ability to scavenge reactive oxygen species, indicating that transgenic tomatoes have higher cold tolerance (Li et al., 2023).

Cold adaptation in maize is mediated by nitric oxide (NO) metabolism regulated by hemoglobin. Under cold stress, the expression of *ZmPgb1.1* is significantly upregulated. The protein encoded by it converts NO into nitrate (NO_3_^-^) through NO dioxygenase activity, reducing the intracellular NO level. The increase of NO inhibits the key enzymes of brassinolide (BR) biosynthesis and the signaling pathway. The induction of *ZmPgb1.1* alleviates this inhibition, promotes cell elongation and division, and enhances the cold tolerance of maize (Mira et al., 2021).

The precise definition of a plant heat sensor was simplified by Ive De Smet. According to this definition, an assumed plant heat sensor must satisfy three criteria: (i) high temperature directly alters the sensor’s activity or conformation; (ii) high temperature modifies the activity and conformation required for temperature signaling; and (iii) changes in its activity and conformation directly influence the plant’s temperature response (Vu et al., 2019). An ongoing scientific question remains whether the cold receptor functions similarly to the high-temperature receptor.

**Figure 2:**
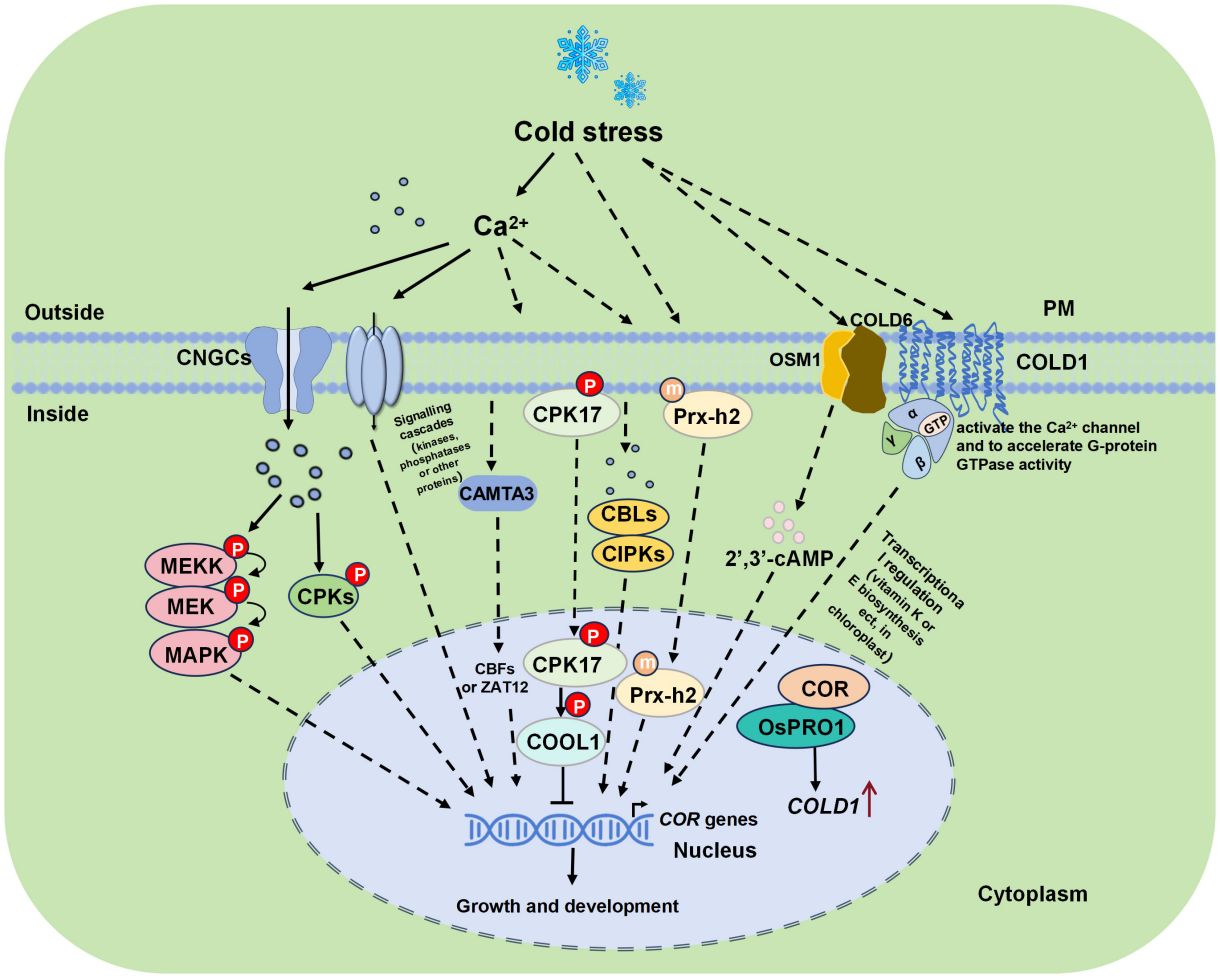
Perception of cold signals in plants. Under cold stress, plasma membrane localized cold sensors, including cyclic nucleotide gated channels (CNGCs) and the rice-specific COLD1/OSM1 complex, which mediate extracellular Ca^2+^ influx, thereby generating stimulus-specific calcium signatures. In the cytosol, calcium-dependent effectors such as calcium-dependent protein kinases (CPKs), CBL-CIPK complexes, and MAPK cascades are activated and propagate signals to the nucleus, where they phosphorylate transcriptional regulators to modulate the expression of cold-responsive genes, including *CORs, COOL1*, and *OsPRO1*. Additional signaling molecules, including peroxiredoxin-h2 (Prx-h2) and 2’,3’-cyclic AMP (2’,3’-cAMP), contribute to cold signal amplification and integration. Collectively, these regulatory layers coordinate cold tolerance with growth and developmental programs through precise spatiotemporal control of cold-responsive gene transcription. Regulatory relationships in the schematic are defined as follows: solid arrows denote activation; T shaped lines indicate repression; dashed lines represent direct or indirect regulation; the blue double-stranded DNA depicted in the figure corresponds to the genomic DNA template for transcription of the gene.

The cold regulatory network of plants is complex and efficient (**Figure 2**). As the primary barrier against external environmental stresses in plants, the structural and functional stability of the cell membrane is severely challenged under cold stress. Cold conditions markedly modify the composition of membrane lipids, as well as the activity and conformation of membrane proteins, consequently influencing membrane fluidity and structural integrity (Chen et al., 2021; Gao et al., 2024a). These physicochemical alterations initiate a cascade of intricate signal transduction pathways in plants, leading to the activation of adaptive response mechanisms. Recent studies have demonstrated that in maize, several genes involved in glyceride metabolism, *KASII*, *ACP2*, *Ms33*, and *SAD2*, which positively contribute to enhanced cold tolerance. Under cold stress, the maize *kasII* mutant not only displays increased leaf damage but also shows aggravated ion leakage, exhibiting a cold-sensitive phenotype. Lipid metabolism modulates the proportion of unsaturated fatty acids in the cell membrane, which in turn alters its phase transition temperature and fluidity, allowing maize to maintain normal membrane morphology under cold conditions (Gao et al., 2024a).

Ca^2+^-permeable channel CYCLIC NUCLEOTIDE-GATED CHANNEL20 (CNGC20), as a core component, positively regulates the freezing tolerance of *Arabidopsis thaliana* by mediating cold-induced Ca^2+^ influx (Peng et al., 2024). The hypersensitive phenotypes of the *camta3* single mutant and the *camta1xcamta3* double mutant to cold stress confirm the crucial role of the CAMTA family in cold acclimation. Notably, the temperature-responsive activity of CAMTA3 is independent of its C-terminal calmodulin-binding domain, suggesting the existence of other unknown temperature perception mechanisms (Chao et al., 2022). The decoding of calcium signals depends on a complex protein interaction network. In rice, COLD1 interacts with the G-protein α subunit RGA1 to activate the plasma membrane Ca^2+^ channel, leading to a sudden increase in the cytoplasmic Ca^2+^ concentration (Ma et al., 2015). The differential regulation of this pathway is directly related to the differentiation of cold tolerance between japonica rice (cold-tolerant) and indica rice (cold-sensitive). Metabolomic studies further reveal that COLD1 enhances membrane stability and antioxidant defense by regulating the dynamic conversion of the vitamin E-vitamin K1 sub-network (Luo et al., 2021).

The transmission of intracellular Ca^2+^ signals also depends on modular systems such as CBL-CIPK. In maize, the calcium-dependent protein kinase 17 (CPK17) can translocate to the nucleus under cold stress and stabilize the key regulator of maize cold tolerance, the bHLH transcription factor COOL1, negatively regulating cold tolerance. The *COOL1* gene, as a key natural variant for maize cold tolerance and high-latitude adaptation, provides important theoretical basis for maize cold tolerance breeding (Zeng et al., 2025). Overexpression of the *COOL1* gene compromises cold tolerance in maize, whereas targeted knockout of *COOL1* markedly enhances cold tolerance in maize seedlings. Integrated multi-omics analyses demonstrate that COOL1 functions as a negative regulator of cold tolerance by directly repressing the transcription of two critical cold-responsive genes: the master transcription factor *DREB1/CBF* and the trehalose biosynthesis gene *TPS*. Notably, under optimal growth conditions, loss-of-function mutants of *cool1* exhibit no significant alterations in major yield-related agronomic traits. Furthermore, the favorable haplotype COOL1^HapA^ is predominantly enriched in maize germplasm adapted to high-latitude, cold-prone regions of northern China. Collectively, this work identifies COOL1 as a promising molecular target for breeding cold-resilient maize varieties and provides a genetically grounded strategy to enhance maize adaptation to cold environments, which contributing meaningfully to sustainable production and climate resilience in global maize agriculture. This modular signal transmission mechanism allows plants to dynamically adjust their physiological responses according to temperature changes, forming a complete signaling pathway from changes in membrane physical state to gene expression regulation.

**Figure 3:**
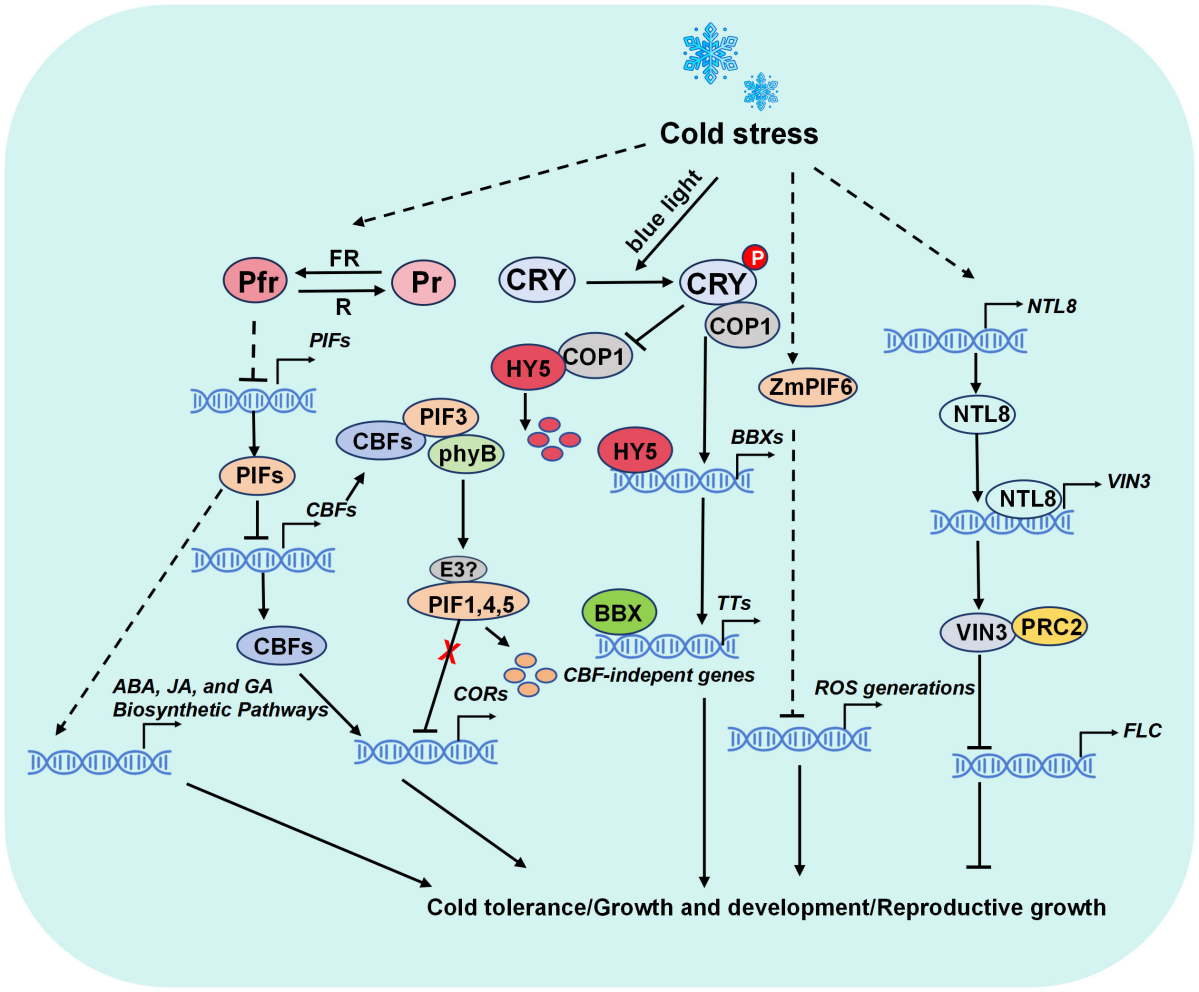
Identification and functional analysis of other potential temperature sensors involved in plant temperature responses. Under cold stress, plants integrate light-sensing pathways, including phytochrome B (phyB)-PIF and cryptochrome (CRY)-COP1-HY5 modules, with chromatin-regulatory complexes such as NTL8-VIN3-FLC to coordinately modulate both CBF-dependent and CBF-independent cold-responsive signaling cascades. This integrated network transcriptionally regulates cold-responsive genes (CORs), reprograms hormone biosynthesis, and maintains ROS homeostasis, thereby balancing cold tolerance with growth, development, and reproductive fitness. The schematic thus provides a mechanistic framework for elucidating cross-talk between light and temperature signaling pathways. Regulatory relationships are denoted as follows: solid arrows indicate activation; T shaped lines indicate repression; dashed lines denote direct or indirect regulation; the blue double-stranded DNA depicted in the figure corresponds to the genomic DNA template for transcription of the gene; and the “×” symbol signifies functional inhibition or blockade.

In plants, additional thermoreceptors may exist to modulate flowering and growth (**Figure 3**). Phytochrome B (PhyB), a red light photoreceptor, is critically involved in temperature sensing and the regulation of plant growth and development (Jung et al., 2016). This protein undergoes a photoreversible transition between two conformational states: the red light-activated Pfr (far-red light-absorbing) form and the dark-adapted Pr (red light-absorbing) form. Importantly, the rate of dark reversion (Pfr→Pr) of PhyB increases with ambient temperature, allowing it to integrate both light and thermal signals (Klose et al., 2015). Furthermore, through the PIF3-CBF (C-REPEAT BINDING FACTOR) module, PhyB contributes to the enhancement of freezing tolerance by activating the COLD-REGULATED (COR) gene network, thereby improving plant survival under freezing stress (Jiang et al., 2017, Jiang et al., 2020a). Under cold condition, enhanced PhyB activity suppresses PIF4 function and coordinates photomorphogenesis (Klose et al., 2015). This mechanism reveals the cross-regulatory network between the light signaling pathway and cold adaptation. Such as in Maize, heterologous overexpression of *ZmPIF6* in rice significantly improves cold tolerance in transgenic lines, likely by reducing reactive oxygen species accumulation and enhancing cell membrane stability, thereby mitigating physiological and biochemical damage under cold stress. Additionally, overexpression of *ZmPIF6* increases grain length and width, leading to an overall enlargement of grain size. This effect is attributed to the regulatory function of ZmPIF6 on genes associated with both cold tolerance and grain development in rice (Li et al., 2024b). Interestingly, recent findings demonstrate that CRY2 is critical for cold acclimation under blue light conditions. Cold treatment could stabilizes the blue light-induced phosphorylated form of CRY2 (Li et al., 2021). Exposure to blue light can simulate cold stress, indicating a close correlation between light quality and temperature. In tomatoes, the knockout of SlPIF4 increased cold sensitivity, whereas overexpression of SlPIF4 enhanced cold tolerance. SlPIF4 not only directly binds to the promoter of *SlCBF* gene to activate its expression but also modulates the biosynthesis and signaling of phytohormones, including abscisic acid, jasmonic acid (JA), and gibberellin (GA), in response to low temperatures. Furthermore, SlPIF4 directly activates the *SlDELLA* gene (GA-INSENSITIVE 4, SlGAI4) under cold stress, and SlGAI4 positively regulates cold tolerance. Additionally, SlGAI4 suppresses the accumulation of SlPIF4 protein, thereby establishing multiple coherent feedforward regulatory loops (Wang et al., 2020).

In the regulation of flowering time, the NAC-type transcription factor NTL8 mediates cold signals within the vernalization pathway. Under cold conditions, NTL8 protein gradually accumulates via ubiquitination, leading to upregulated transcription of VERNALIZATION INSENSITIVE 3 (VIN3) (Antoniou-Kourounioti et al., 2018). As a central vernalization component, VIN3 recruits the histone demethylase complex Polycomb Repressive Complex 2 (PRC2) to suppress expression of the floral repressor FLOWERING LOCUS C (FLC), thereby promoting flowering (Sung and Amasino, 2004). This slow, temperature-responsive regulatory mechanism ensures that plants transition to reproductive growth only after prolonged cold exposure. thereby repressing plant flowering (Kumar et al., 2012).

In cash crops and horticultural plants, cold stress not only affects the growth and development process, but impacts the ornamental or edible value in aspects such as fruit development, flowering regulation, and post-harvest storage. During the fruiting period of tomatoes, cold stress inhibits the process of sucrose transport from leaves to the shoot apex, resulting in abnormal fruit development. In *STP2* overexpressing plants, the accumulation of glucose and arabinose enhances the arabinosylation of CLV3, restoring the inhibitory effect on WUS, thereby alleviating the multi-locular malformation induced by low temperature (Li et al., 2025).

For horticultural crops, such as apple trees, the physiological and developmental responses to environmental and management factors are well documented in the literature. Apple trees require a long period of cold in winter to bloom and grow in the following spring. The release of dormancy and bud germination in spring require the silencing of *MdDAM1*. However, the silencing of *MdDAM1* will lead to defects in the normal phenomenon of growth cessation in autumn, thus affecting flower bud development and fruit-setting (Moser et al., 2020). When apples are exposed to low temperatures for a long time after being picked, it may lead to discoloration of the peel and even the appearance of necrotic patches. However, sorbitol can act as a cryoprotectant. High expression of *MdS6PDH* will increase the sorbitol content in the fruit, thus playing a protective role. Heterologous expression of *MdS6PDH* in *Arabidopsis thaliana* also confirms its involvement in the cold stress process (Busatto et al., 2018).

Collectively, these studies elucidate a sophisticated regulatory network that enables plants to integrate light and temperature cues through multi-tiered molecular mechanisms, ranging from signal perception by membrane receptors, through transcriptional cascades orchestrating gene expression, to epigenetic memory shaped by chromatin remodeling. This integrated framework forms a cohesive system underlying environmental adaptation. Future research should focus on deciphering the synergistic and antagonistic interplay among these signaling pathways, as well as how evolutionary processes have refined these mechanisms to foster adaptation across diverse ecological niches.

## 4 Cold signal regulatory network of plants

Dehydration-Responsive Element Binding protein 1 (DREB1s) are members of the APETALA2/Ethylene Responsive Factor (ERF) superfamily and are widely distributed in higher plants. Research has demonstrated that a range of plant species, including *Arabidopsis thaliana*, *Triticum aestivum* L., *Zea mays* L., and *Oryza sativa* L., possess this class of transcription factors (Badawi et al., 2007; Ito et al., 2006; Qin et al., 2004; Stockinger et al., 1997). Typical downstream targets include COR genes such as RESPONSE-TO-DEHYDRATION 29A (RD29A), COLD-RESPONSIVE 15A (COR15A), Ethylene-responsive factor 54 (ERF54) and KIN1. Under cold stress, DREB1s become activated and specifically recognize the conserved CCGAC motif within the promoters of these target genes, thereby initiating transcription. This activation subsequently leads to the expression of functional proteins, including antifreeze proteins and enzymes involved in osmotic adjustment, ultimately improving plant tolerance to low temperatures (Chen et al., 2024; Chen et al., 2025a; Gilmour et al., 1998; Jaglo et al., 2001; Kamble, 2024; Stockinger et al., 1997; Zhang et al., 2022).

Cold acclimation studies have demonstrated that the functional roles of DREB1 family members involve complex regulatory mechanisms. Single mutants of *dreb1c* or *dreb1a* displayed only a mild freezing-sensitive phenotype following cold treatment, whereas the *dreb1b* mutant showed increased freezing tolerance (Novillo et al., 2004, Novillo et al., 2007). Microarray analyses further indicated that over 100 genes are co-regulated by CBF1, CBF2, and CBF3 (Gilmour et al., 2004), underscoring the high degree of functional redundancy within this gene family. However, when CBFs are overexpressed under the control of the 35S promoter, ectopic gene expression occurs, resulting in non-specific binding of CBFs to their target genes. This suggests that the expression of *DREB1s* requires precise spatiotemporal regulation.

To further elucidate the biological functions of DREB1s, researchers generated *cbfs* triple mutants using CRISPR/Cas9-mediated gene editing. Phenotypic analyses revealed that the triple mutants exhibited pronounced freezing sensitivity, providing direct evidence for the central role of DREB1s in plant cold responses. By integrating ChIP-Seq data, researchers further identified the target genes of CBFs and demonstrated that their regulatory networks encompass multiple physiological processes, including hormone signaling, light response pathways, and circadian regulation (Jia et al., 2016; Song et al.2021; Zhao et al., 2016). Such as, transcriptomic analysis based on RNA sequencing revealed that a total of 134 genes are regulated by CBF, among which 112 genes are upregulated and 22 are downregulated (Zhao et al., 2016). Similarly, transcriptome profiling analysis reveals that CBF genes regulate 414 cold-responsive (COR) genes, among which 346 are CBF-activated genes and 68 are CBF-repressed genes. The findings demonstrate that CBF proteins are extensively involved in the regulation of carbohydrate and lipid metabolism, cell wall modification, and gene transcription. This fully revealing the important status of DREB1s as hub factors in stress responses and plant growth and development.

Besides, ICE1, a key member of the bHLH transcription factor family, plays a central role in the transcriptional regulation of *DREB1s*. It has been demonstrated that ICE1 specifically binds to the G-BOX element within the promoter region of its target gene *DREB1A*, thereby activating transcription and enhancing *DREB1A* expression. Functional analyses reveal that the *ice1* loss-of-function mutant displays a pronounced cold-sensitive phenotype, whereas transgenic lines overexpressing ICE1 exhibit enhanced freezing tolerance. Transcriptional profiling further indicates that *DREB1A* expression is significantly reduced in the *ice1* mutant compared to the wild type. Similarly, loss of function in its homolog ICE2 leads to marked downregulation of *DREB1C* expression, accompanied by a freezing-sensitive phenotype in the *ice2* mutant. These findings collectively demonstrate that ICE1 and ICE2 are functionally conserved and act as positive regulators in the plant cold response pathway (Cheong et al., 2003; Chinnusamy et al., 2003; Ding et al., 2015). Recent studies demonstrate that the activation of ICE1/SCRM transcription factors through phosphorylation by MAPK3/6 promotes the establishment of zygote polarity in *Arabidopsis thaliana* (Chen et al., 2025a). In maize, ZmICE1a acts as a negative regulator of IAA and JA biosynthesis, indirectly modulating defense responses in the peripheral endosperm. Furthermore, ZmICE1a interacts with ZmJAZ9 and contributes to the regulation of the JA signaling pathway by influencing the expression of the defense-related gene MPI (Wang et al., 2024). Overexpression of *OsPIN5b* in rice leads to a significant increase in plant height but reduces cold tolerance (Fu et al., 2025).

Plant hormones are indispensable for the transcriptional regulation of *DREB1s* genes. The BR signaling pathway is closely associated with the cold response. Under cold stress, the dephosphorylated form of BZR1 protein accumulates and binds to conserved E-box/BRRE motifs in the promoters of downstream target genes, such as *CBF1*, thereby enhancing the plant’s cold tolerance (Li et al., 2017a). BR and JA act synergistically to regulate cold tolerance in apple (*Malus*×*domestica*) through the JAZ-BIM1-CBF module (An et al., 2023a). Cold stress elevates BR levels and promotes the accumulation of BZR1. Overexpression of BR biosynthesis genes such as *DWARF* (DWF) and *BZR1* upregulates the abscisic acid biosynthesis gene *NCED1*, thereby increasing ABA levels and enhancing tomato tolerance to cold (An et al., 2023b). In addition, BZR1 regulates plant cold response through a CBF-independent pathway, as evidenced by its involvement in modulating cold-responsive genes such as *WRKY6*, *PYL6*, *SOC1*, *SAG21*, and *JMT*. Research results demonstrate that BZR1 specifically binds to the E-box in the promoter region of *WRKY6*, thereby positively regulating freezing tolerance in *Arabidopsis thaliana* (Li et al., 2017b).

Studies have shown that heterologous expression of *Arabidopsis CBF1* in tomato causes dwarfism, while exogenous application of gibberellins (GAs) restores the wild-type phenotype. Further investigation revealed that CBF1 downregulates GA levels by upregulating transcripts of GA2 oxidase genes, leading to accumulation of the DELLA protein RGA and consequently retarded plant growth. Moreover, the accumulated DELLA protein significantly enhances CBF1-mediated cold acclimation and freezing tolerance (Agarwal et al., 2006; Hsieh et al., 2002; Magome et al., 2004). ABA activates the *CRT* promoter element and induces the cold response process, accompanied by an increase in CBF protein levels (Knight et al., 2004). Overexpression of the *Arabidopsis* freezing-tolerance gene AtICE1 in indica rice resulted in transgenic lines that, under cold stress conditions, exhibited significantly lower accumulation of MDA and H_2_O_2_, higher membrane stability, and increased seedling survival rate compared to wild-type plants. Concurrently, the expression levels of *OsDREB1A*, *OsMYB3R2*, and *OsTPP1* were markedly upregulated in the transgenic lines. Under non-stress conditions, the *AtICE1*-overexpressing lines showed significantly higher stomatal density and stomatal conductance, along with superior performance in water-use-related parameters such as stomatal conductance, photosynthetic efficiency, and instantaneous water use efficiency relative to the wild type. These results demonstrate that AtICE1 confers multiple stress tolerance in indica rice, and support the functional conservation of *ICE1* genes across plant species in regulating stress tolerance and stomatal development (Verma et al., 2020). Overexpression of the *Arabidopsis ICE1* homolog *ZmICE1* in maize significantly enhances cold tolerance, whereas *zmice1* knockout lines exhibit pronounced cold-sensitive phenotypes. Further investigation confirms that ZmICE1 enhances maize cold tolerance by directly and positively regulating the expression of cold-responsive genes *ZmDREB1s*, indicating the evolutionary conservation of the ICE1-CBF regulatory module in maize (Jiang et al., 2020a). The transgenic *Arabidopsis* lines overexpressing *TaICE41* and *TaCBFIVd-B9* demonstrated markedly enhanced freezing tolerance, exhibiting higher survival rates and proline content compared to the wild-type. Similarly, transgenic barley lines expressing *TaCBF14* and *TaCBF15* displayed superior freezing resistance relative to the wild-type. The aforementioned results demonstrate the functional conservation of the ICE1-CBF-COR pathway in the regulation of freezing tolerance in barley and wheat (Guo et al., 2019; Jung et al., 2025). JA, acting upstream in the ICE-CBF signaling pathway, positively regulate freezing tolerance in *Arabidopsis* (Hu et al., 2013). In contrast, ethylene (ET) reduces cold tolerance by suppressing the CBF/DREB1 pathway in *Arabidopsis* and soybean (Robison et al., 2019; Shi et al., 2012), highlighting the diversity and complexity of hormonal regulation in plant cold responses.

GABA functions as a novel long-distance signal that enhances cold tolerance in grafted cucumber seedlings with figleaf gourd rootstock, a process achieved through the modulation of CBF-signaling pathways, antioxidant system, and stomatal aperture (Qin et al., 2024).

Phosphorylation of protein kinases represents a pivotal post-translational modification that regulates the transcriptional activity of downstream target genes. The MAPK6 signaling pathway is critically involved in transcriptional regulation. Specifically, MAPK6 phosphorylates Ser^168^ of the cold-signaling transcriptional repressor MYB15, a modification that diminishes MYB15’s binding affinity to the *CBF3* promoter (Kim et al., 2017). Furthermore, ICE1 interacts with MYB15 and suppresses its DNA-binding activity, thereby promoting the upregulation of *CBFs* expression. The E3 ligases PUB25 and PUB26 fine-tune the cold response by dynamically modulating the stability of ICE1 and MYB15 proteins through the addition of distinct polyubiquitin chains (Wang et al., 2019, Wang et al., 2023b).

In *Arabidopsis thaliana*, cold stress specifically activates the kinase activity of the receptor-like cytoplasmic kinase cold-responsive protein kinase 1 (CRPK1). Upon activation, CRPK1 phosphorylates plasma membrane-localized 14-3–3 proteins, triggering their translocation into the nucleus. Within the nucleus, the 14-3–3 proteins interact with DREB1C/DREB1A and mediate their degradation via the 26S proteasome pathway, ultimately reducing the freezing tolerance of *Arabidopsis thaliana* (Liu et al., 2017). This mechanism illustrates how phosphorylation-mediated control of protein localization and stability negatively regulates the cold response pathway. The plasma membrane-localized Ca^2+^/calmodulin-regulated receptor-like kinases CRLK1 and its homolog CRLK2 positively regulate the expression of *CBF* and *COR* by phosphorylating MEKK1 and enhancing its activity, thereby promoting the cold acclimation process in plants (Yang et al., 2010a, Yang et al., 2010b; Zhao et al., 2017). In addition, the SNF1-related protein kinase (SnRK2s) family plays a key role in cold acclimation. Among them, SnRK2.6/OST1 can phosphorylate the β-subunits of NAC, BTF3 (basic transcription factor 3) and BTF3-like (BTF3L). Phosphorylated BTF3 interacts with CBF proteins to stabilize the protein activity of CBF and inhibit its ubiquitination and degradation, thus enhancing the freezing tolerance of *Arabidopsis* (Ding et al., 2018). In contrast, MAPK3/6 phosphorylates ICE1 under cold stress, inhibits the transcription of the *ICE1*, down-regulates the expression of *CBFs*, and plays a negative regulatory role in plant freezing tolerance (Li et al., 2017a; Zhao et al., 2017). These studies indicate that phosphorylation modifications achieve bidirectional and fine regulation of CBF-dependent signal transduction by targeting different signaling nodes.

Ubiquitination is a central regulatory mechanism governing protein stability. As a key E3 ubiquitin ligase, high expression of OSMOTICALLY RESPONSIVE GENE 1 (HOS1) enables its interaction with ICE1, leading to ICE1 ubiquitination. This modification facilitates ICE1 degradation via the 26S proteasome pathway, thereby reducing its stability under cold conditions. Previous studies have demonstrated that *HOS1* overexpression significantly suppresses *CBF3* expression and consequently diminishes plant freezing tolerance (Dong et al., 2006). Low temperature also activates the OST1-HOS1 module, which promotes the ubiquitination and degradation of HAT1, leading to the induction of *CBF* and *COR* gene expression and enhancing plant cold tolerance (Kang et al., 2025).

In contrast, SUMOylation mediated by the SUMO E3 ligase SIZ1 exerts an antagonistic effect. SIZ1 catalyzes the conjugation of SUMO to substrate proteins, resulting in ICE1 SUMOylation. This modification attenuates the extent of HOS1-induced polyubiquitination, enhances ICE1 stability, and thereby improves freezing tolerance in *Arabidopsis* (Miura et al., 2007). The dynamic balance between HOS1-mediated ubiquitination and SIZ1-mediated SUMOylation maintains homeostasis of ICE1 protein levels, ensuring precise regulation of the CBF-dependent signaling pathway. Overexpression of SIZ1 attenuated the inhibitory effect of ABA on root growth, and the overexpression lines exhibited enhanced freezing tolerance compared to the wild type under both cold acclimation and non-acclimation conditions (Miura and Nozawa, 2014).

Studies indicated that PRC1 and the histone variant H2A.Z act cooperatively to reset cell fate under low temperature. PRC1 initiates repression of embryonic developmental programs by catalyzing H2A.Z ubiquitination (H2A.Zub), while PRC2 sustains this repression via H3K27me3 marking. The transcription factor TOE1 interacts with the INO80-C complex to evict H2A.Z; cold stress suppress *TOE1* expression, disrupt H2A.Z removal, and promote accumulation of PRC1-H2A.Z, which in turn represses gene expression and reprograms cell identity (Kumar and Wigge, 2010). Additionally, the histone methyltransferase SDG7 directly interacts with SWC6, a subunit of the SWR1 complex, facilitating the deposition of H3K36me3 marks and the deposition of the histone variant H2A.Z at the FLC locus.

As a key regulator of flowering, GI (GIGANTEA) also plays a significant role in plant responses to cold stress. Cold stress induces *GI* expression, and the *gi1–3* mutant exhibits markedly reduced freezing tolerance. However, the expression of CBFs remains unaltered, indicated that GI positively regulates the freezing tolerance of *Arabidopsis thaliana* through CBF-independent pathway (Cao et al., 2005). Under cold conditions, GI interacts with HOS15 and HD2C to form a repressor complex that suppresses CBF binding, thereby inhibiting the activation of *COR* genes (Ji et al., 2025). Physiological analysis of the *HsfA1d* (Heat Shock Transcription Factor A1d) mutant under cold conditions revealed that HsfA1d functions as a positive regulator of hypocotyl elongation during cold stress. It significantly enhances the expression of numerous ribosome biogenesis genes under cold stress. For instance, HsfA1d could directly binding to the promoter regions of ribosomal protein genes *RPL9* and *RPL18*, thereby promoting their transcription. Phenotypic evaluation of ribosomal protein gene mutants under cold stress demonstrated that their mutations markedly suppress hypocotyl growth, confirming that HsfA1d positively regulates plant growth at low temperatures via a CBF-independent pathway (Liu et al., 2021).

**Figure 4:**
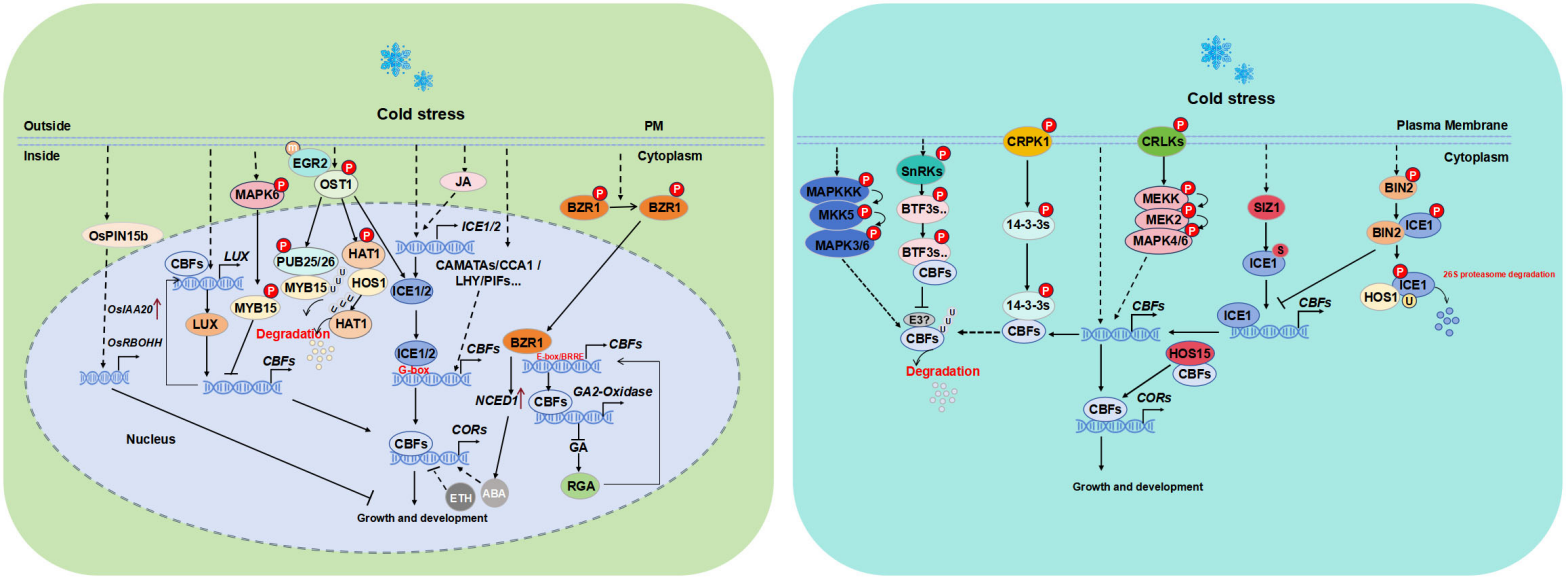
Regulation of *DREB1* gene expression at the transcriptional (left) and post-translational level (right). When plants are exposed to cold stress, upstream components can perceive external low-temperature environmental signals in a timely manner, and activate relevant intracellular signal transduction components, such as phosphorylation modification and ubiquitination modification of proteins, thereby activating the expression of related genes in the cold signal transduction pathway and regulating plant growth and development under low-temperature conditions. Regulatory relationships are denoted as follows: solid arrows indicate activation; T shaped lines indicate repression; dashed lines denote direct or indirect regulation; the blue double-stranded DNA depicted in the figure corresponds to the genomic DNA template for transcription of the gene.

The DUF231 family protein encoded by *ESK1* (ESKIMO1) plays a distinct role in the cold response. Under cold conditions, the *esk1* mutant exhibits a freezing-tolerant phenotype due to the accumulation of high levels of the protective solute proline (Xin et al., 2007). In contrast, the *sfr6* (SENSITIVE TO FREEZING 6) mutant is hypersensitive to low temperature. In *sfr6* mutants, cold-induced expression of canonical COR (cold-regulated) genes is severely attenuated, whereas basal and cold-induced transcript accumulation of *CBF1*, *CBF2*, and *CBF3* remains unaffected. Notably, ectopic overexpression of *CBF1* or *CBF2* in the *sfr6* background fails to restore robust induction of *COR* genes, which is a response consistently observed in wild-type plants under identical conditions, indicating that SFR6 functions downstream of CBF transcription factors and is indispensable for the transcriptional output of the CBF regulon (Knight et al., 1999, Knight et al., 2009). *Arabidopsis thaliana* cold shock domain protein 3 (AtCSP3) shares a cold shock domain with bacterial cold shock proteins (CSPs) and is involved in the acquisition of freezing tolerance in plants. The AtCSP3 protein localizes to both the nucleus and the cytoplasm. Compared to wild-type plants, *atcps3* knockout mutants (*atcsp3-2*) exhibit heightened susceptibility to freezing injury under both non-acclimated and cold-acclimated conditions, whereas the expression of *CBF* and its downstream genes remains unaltered in *atcsp3–2* mutants during cold acclimation. Overexpression of *AtCSP3* in transgenic plants confers superior freezing tolerance relative to wild-type plants. These findings indicate that AtCSP3 participates in regulating Arabidopsis freezing tolerance through a pathway independent of the CBF signaling cascade (Kim et al., 2009). Apple trees are susceptible to cold stress. Studies have identified MYB88 and its homolog FLP (MYB124), which function as R2R3 MYB transcription factors (TFs), as playing a potential role in cold stress responses in both apple and *Arabidopsis*. MdMYB88/MdMYB124 can directly regulate the cold shock domain protein 3 (MdCSP3). Furthermore, MdMYB88 and MdMYB124 promote anthocyanin accumulation and hydrogen peroxide detoxification under cold stress. These findings indicate that MdMYB88 and MdMYB124 positively modulate cold tolerance and the expression of cold-responsive genes through a CBF-independent pathway (Xie et al., 2018). The *HOS9* (High Expression of Osmotically Responsive Genes 9) gene encodes a homeobox-class transcription factor. The *hos9* mutant exhibits a freezing-sensitive phenotype irrespective of cold acclimation, while the expression of *CBF* genes remains unaltered in the mutant. Microarray data analysis reveals that genes affected by the *hos9–1* mutation are not regulated by the CBF family. These results indicate that HOS9 plays a critical role in plant growth and development as well as partial freezing tolerance, and its mechanism of action is mediated through the regulation of gene activities independent of the CBF pathway (Zhu et al., 2004). AtSF1 contributes to cold adaptation by facilitating proper intron splicing of nuclear genes involved in chloroplast development, such as *PRPL28* and *PRPL4*, thereby ensuring chloroplast functionality under cold stress (Zhu et al., 2020). This redox-based regulatory mechanism offers a novel perspective on how plants perceive and respond to cold stress (**Figure 4**).

The regulatory network mediated by CBF is a classic pathway for cold response in angiosperms. It not only exists in model plants but also widely exists in food crops and economically important cash crops. According to phylogenetic studies, it is highly likely that the CBF regulatory network is the same evolutionary choice made by plants to adapt to ancient global cooling (Nie et al., 2022). However, plants of different species still have their own characteristic cold perception pathways. For example, the *COOL1* is a key cold-responsive factor specifically identified and intensively studied in maize. As a transcriptional repressor, it negatively regulates the cold tolerance of maize through the regulation mediated by HY5 and CPK17, and its natural variation is an important genetic basis for maize to adapt to the low-temperature environment at high latitudes (Zeng et al., 2025).

## 5 Enhancing cold tolerance in crops via genetic engineering

**Figure 5:**
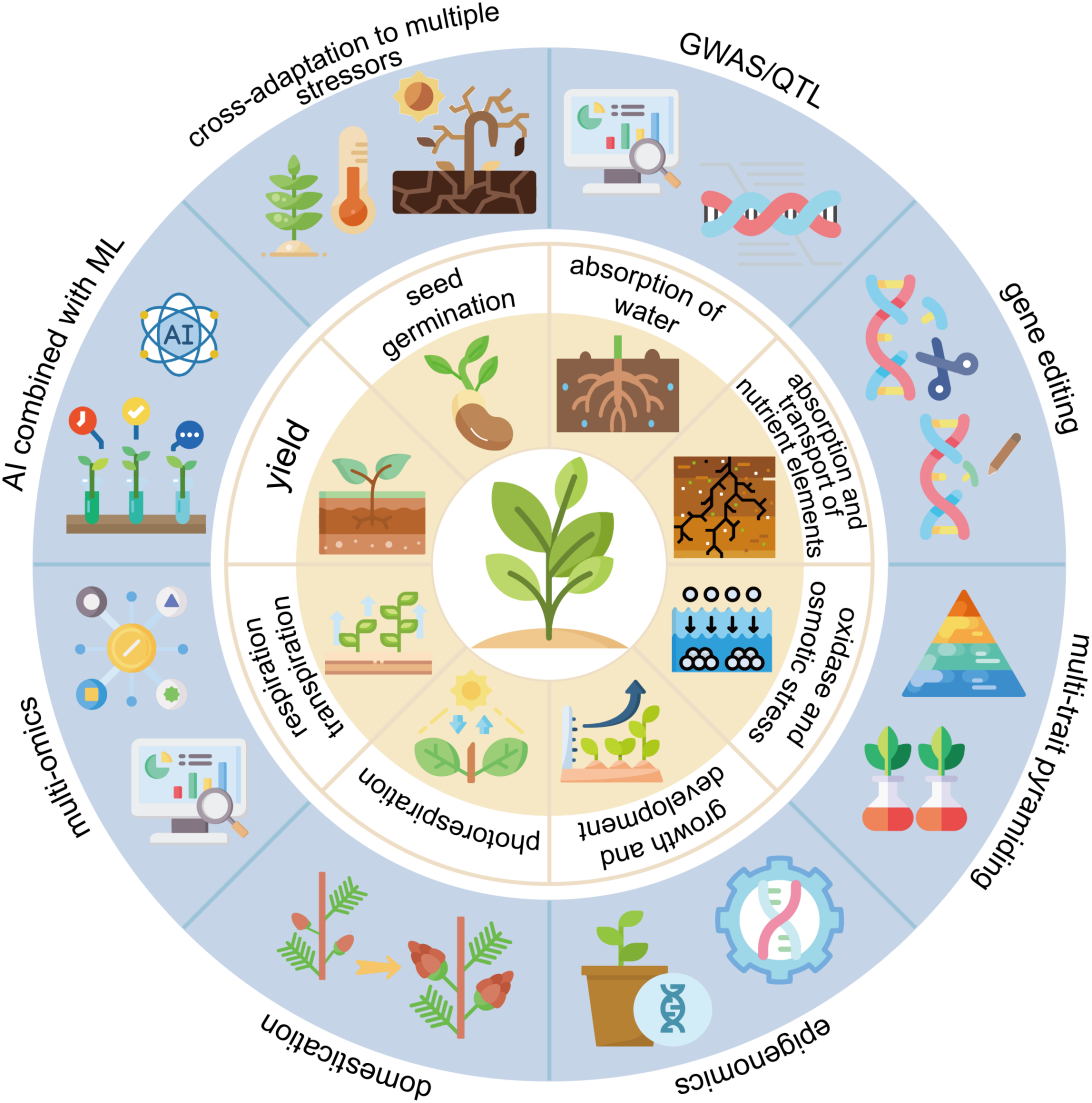
Methods for improving the cold tolerance of plants. (Yellow circle) Cold stress exerts differential effects on the vegetative and reproductive growth of plants. (Blue circle) Integration of gene editing and multi-omics technologies for genetic breeding improvement to cultivate cold-tolerant, high-yield, and high-quality crops.

In the context of global climate change, the application of cold response mechanisms in crop breeding has become a critical frontier for enhancing agricultural resilience. Here, we synthesize the translational advancements in this field, highlighting tangible breakthroughs and emerging strategies (**Figure 5**).

### 5.1 Commonly employed genome editing technologies in crop improvement

Genome editing is a powerful biotechnological tool enabling precise modification of any gene. Key tools include ZFNs, TALENs, CRISPR/Cas9, mitoTALEN, prime editing, and RLR, widely applied to develop crops resistant to abiotic and biotic stresses. ZFNs and TALENs both use customizable DNA-binding domains (zinc fingers or TALE repeats) fused to the FokI nuclease, requiring dimerization for double-strand break induction. Their reliance on protein, DNA interactions makes design complex, costly, and less efficient compared to CRISPR-based systems. Consequently, ZFNs and TALENs face major practical limitations in scalability and adoption (Becker and Boch, 2021; Kumari et al., 2025; Venezia et al., 2021; Xu et al., 2019; Zhu and Zhu, 2022). Genome editing offers several benefits over earlier editing tools with a significant advantage of having the ability to precisely target and mutate individual genes across the entire plant genome. The CRISPR technology uses a simpler, more versatile and precise method of mutagenesis, facilitating the transmission of desired traits to offspring (Wu et al., 2024). This approach can induce mutations at a specific location within the targeted gene, enhancing the significance of plant characteristics (Gupta et al., 2023a, Gupta et al., 2023b).

Mitochondrial genome editing in plants is feasible using mitoTALENs, TALENs fused with mitochondrial targeting signals, which is to achieve stable, site-specific modifications. Key successes include editing of the mitochondrial atp6–1 gene in *Arabidopsis*, rice, and rapeseed without affecting its nuclear counterpart. Optimization studies identified the *RPS5A* promoter and conventional TALEN architecture as most effective for editing efficiency. MitoTALENs (and newer mitoTALECD) enable targeted deletions or stop-codon insertions in mitochondrial genes (e.g., orf125 in potato), though phenotypic effects remain subtle in vegetatively propagated crops. Despite their precision, TALE-based tools face practical limitations due to labor-intensive assembly, highlighting the need for more scalable organelle-editing platforms such as CRISPR/Cas derivatives (Arimura et al., 2020; Nicolia et al., 2024; Yang et al., 2022).

The CRISPR/Cpf1 (Cas12a) system is a versatile genome editing platform that uses a single crRNA, features compact size, and generates staggered DNA ends, enabling more precise insertions and potentially favoring HDR over non-homologous end joining (NHEJ). It recognizes T-rich PAM sequences (e.g., TTTV), distinct from Cas9’s NGG, and its RuvC-only nuclease domain lacks the HNH domain, contributing to different cleavage mechanics. Cas12a has been successfully applied in major crops, which including rice, *Arabidopsis*, tobacco, soybean, cotton, and maize, demonstrating broad species compatibility and robust site-specific mutagenesis. Its smaller size facilitates delivery via viral vectors or other non-transgenic methods, enhancing translational potential. However, Cas12a exhibits greater temperature sensitivity than Cas9, necessitating engineering for optimal activity in cool-climate crops (Bayat et al., 2018; Lee et al., 2019; Zhu and Zhu, 2022).

RLR is a novel, strand-preserving genome editing tool developed at Harvard that enables simultaneous, high-throughput editing of millions of DNA sequences without inducing double-strand breaks. Unlike CRISPR-Cas9, which relies on error-prone DNA repair and poses off-target risks, RLR uses reverse transcriptase (RT) and single-stranded annealing proteins (SSAP) to generate and integrate mutation-carrying ssDNA during DNA replication. It “barcodes” edited cells, enabling pooled screening and large-scale functional genomics analysis. RLR operates without cleaving genomic DNA, thereby improving cell viability and editing fidelity. Its multiplex capability and scalability make it especially valuable for constructing complex mutant libraries in diverse cell types (Kumari et al., 2025).

### 5.2 Molecular design breeding: precision gene editing

The identification of key cold-responsive genes has facilitated precision breeding approaches. For example, the COLD6-OSM1 complex in rice was found to initiate 2’,3’-cAMP signaling, thereby conferring cold tolerance (Luo et al., 2024). Through marker-assisted selection (MAS), introgression of the japonica COLD6 allele into indica varieties resulted in enhanced chilling resilience. Similarly, the OsSRO1c-OsDREB2B module, which activates cold-responsive genes via liquid-liquid phase separation, represents a promising target for genetic improvement in rice (Hu et al., 2024). In potato, CRISPR-mediated editing of ScF3’H shifted flavonoid biosynthesis toward more hydroxylated compounds such as quercetin, markedly increasing freezing tolerance (Bao et al., 2025). CRISPR/Cas9 has been widely applied in crops to simultaneously enhance yield and improve resistance to biotic and abiotic stresses. It effectively confers disease resistance and tolerance to environmental challenges, such as cold stress, through precise genome editing. Studies confirm its utility in generating non-transgenic edited plants, avoiding regulatory and public concerns associated with transgenic approaches. Its versatility, accessibility, and precision make it a key strategy for climate-resilient crop development. Ultimately, CRISPR/Cas9 contributes significantly to global food security under changing climatic conditions (Hamdan et al., 2022; Mo et al., 2020). CRISPR-mediated knockout of OsNAC050 in rice enhances cold tolerance in seedlings by reducing ROS accumulation and boosting antioxidant enzyme activities (SOD, POD, CAT). Multiplex CRISPR editing of three genes, OsPIN5b, GS3, and OsMYB30, generated triple mutants with improved panicle length, grain size, cold tolerance, and yield. Under 4°C cold stress, the triple mutants showed markedly higher survival rates (70.8% and 79.1%) compared to wild-type rice (45.8%) (Wang et al., 2023b; Yarra and Wei, 2021). As a “search-and-replace” genome editing method, prime editing guided by a programmable prime editing guide RNA (pegRNA) for target localization. PpegRNA comprises a spacer sequence, a scaffold sequence, a reverse transcription template (RTT) containing the edit site, and a primer binding site (PBS), which is capable of not only binding to the target DNA region for desired editing but also precisely directing the Cas9-reverse transcriptase fusion protein to nick a single strand of DNA at the target site and subsequently synthesize DNA containing the correct sequence. In this system, the Cas9 nickase introduces a single-strand break in the DNA guided by the spacer sequence within the pegRNA. The PBS provides a primer binding site for reverse transcriptase, enabling the enzyme to initiate reverse transcription using the subsequent editing template sequence on pegRNA as a template. This process facilitates the direct polymerization of the target edited sequence onto the nicked DNA strand (Anzalone et al., 2019). Achieve high-precision execution across a diverse range of editing tasks and rarely induces DNA alterations at off-target genomic sites. Prime editing enables a broad spectrum of genetic modifications, including all 12 types of single-base substitutions, multi-base substitutions, small insertions (originally up to several dozen nucleotides in the PE3 system), small deletions (originally up to several hundred nucleotides in the PE3 system), and combinations thereof. Furthermore, it can introduce changes at least 33 bp away from the initial nick site generated by prime editing. This flexibility makes prime editing less constrained than base editing or Cas nuclease-mediated HDR, the latter of which requires a PAM sequence in close proximity to the target editing site. Base editing is a precision technique for single-base substitution, enabling the correction of point mutations without inducing double-strand breaks. This system employs a fusion protein comprising a Cas9 nickase and a deaminase enzyme, guided by a single-guide RNA. The Cas9 nickase cleaves only one strand of the DNA double helix, thereby preventing the activation of the error-prone NHEJ pathway. Upon binding, the deaminase enzyme catalyzes chemical modifications of individual DNA bases on the exposed strand. For instance, cytosine base editors (CBEs) convert cytosine (C) to uracil (U), which cellular repair machinery interprets as thymine (T), facilitating a C-G to T-A conversion. Similarly, adenine base editors (ABEs) convert adenine (A) to inosine (I), which is recognized as guanine (G), resulting in an A-T to G-C transition. Currently, prime editing has found broad applications in the cultivation of crops such as rice, maize, and wheat (Cao et al., 2024; Jiang et al., 2022b; Ni et al., 2023; Qiao et al., 2023; Xu et al., 2021; Zhao et al., 2025). Base editing and prime editing are both precision genome editing tools designed for targeted genomic sequence modifications, such as correcting pathogenic mutations. However, they exhibit distinct characteristics: base editing is particularly suitable for specific single-base conversions (e.g., C-G to T-A transitions) and offers higher editing efficiency with simplified operational procedures. Nonetheless, it may introduce undesired bystander edits at adjacent bases and has a restricted targeting scope. In contrast, prime editing is adept at mediating complex genomic alterations, including frameshift mutations, point mutations beyond base conversions, and small insertions/deletions. While it generally demonstrates superior editing efficiency, the procedure is more operationally intricate. A notable advantage of prime editing lies in its minimal byproduct formation and broad applicability.

By employing prime editing technology, researchers successfully generated homozygous mutations in two acetolactate synthase genes, *ZmALS1* and *ZmALS2*, and produced double mutants carrying W542L and S621I substitutions in these genes. These findings offer a feasible and effective strategy for developing non-transgenic herbicide-resistant maize varieties and provide insights for further optimization of plant prime editing systems (Jiang et al., 2020b). Furthermore, researchers engineered an efficient prime editing tool to precisely integrate a 10-base pair heat shock element (HSE) into the promoters of cell wall invertase genes (CWINs) in elite rice and tomato varieties. This modification enhanced carbon allocation to grains and fruits and increased tomato thermotolerance, while maintaining fruit quality (Lou et al., 2025).

### 5.3 Multi-trait pyramiding: optimizing the balance between resistance and productivity

Plant breeders typically aim to enhance multiple quantitative traits, such as yield, quality, and resistance to biotic and abiotic stresses, simultaneously. However, simultaneous improvement of multiple traits remains challenging and is highly dependent on the choice of breeding materials as well as the genetic and epigenetic relationships among the target traits. Advances in molecular marker technology have made molecular breeding the preferred strategy for targeted crop improvement and product development. Techniques such as MAS, marker-assisted backcrossing, marker-assisted recurrent selection, and gene pyramiding have been successfully employed to incorporate single or multiple genes. Multi-trait selection thus represents a viable and effective approach (Gao et al., 2024b; Matos et al., 2024; Tao et al., 2025; Wang et al., 2025).

Breeding programs are increasingly adopting integrated multi-trait approaches, combining cold tolerance with other key agronomic characteristics to develop more resilient and higher-yielding crop varieties. In rice cultivation, for example, diseases like Blast (caused by *Magnaporthe oryzae*) and bacterial blight (caused by *Xanthomonas oryzae* pv. *oryzae*) present major threats to yield stability. To tackle these issues, marker-assisted backcross breeding was used to introduce multiple resistance genes, Pi9 and Pb1 for Blast resistance, and Xa4, xa13, and Xa21 for bacterial blight resistance, into the high-yielding but susceptible variety BRRI dhan48. The resulting advanced breeding lines not only display significantly improved resistance to both diseases but also achieve higher grain yields than the parental line (Latif et al., 2025). This strategy enables the precise integration of target genes while preserving the favorable genetic background of the recurrent parent.

As a complementary strategy, the soybean cultivar Huaxia 3 (HX3) exemplifies inherent stress adaptation. Although originally developed for cultivation in southern regions, HX3 exhibits significant cold tolerance, a trait atypical for its geographic origin. Physiological and molecular analyses reveal that this tolerance is associated with sustained activation of specific stress-responsive pathways, including glutathione metabolism, which alleviates oxidative damage, and redox regulation mechanisms that help preserve cellular homeostasis under cold stress. The persistent upregulation of these pathways enhances the cultivar’s capacity to endure chilling temperatures, thereby promoting stable performance in environments prone to cold spells (Bukhari et al., 2025).

These examples reflect a broader trend in modern plant breeding: the systematic integration of biotic and abiotic stress tolerance mechanisms with yield-related traits, to develop cultivars that perform reliably across diverse and fluctuating field conditions.

### 5.4 Wild relatives and epigenetic regulation

Wild germplasm represents a valuable reservoir of cold-adaptive alleles, offering a crucial pool of genetic diversity that supports plant survival and adaptation under cold stress. These naturally occurring genetic variants play an essential role in breeding programs designed to improve cold tolerance in crops. Recent research has progressively clarified the molecular mechanisms behind such adaptations. For example, in *Arabidopsis thaliana*, the histone deacetylase AtHDA6 was found to stabilize the AtSK2 protein via post-translational deacetylation. This stabilization enhances shikimate metabolism, which is a central pathway responsible for synthesizing aromatic amino acids and various secondary metabolites. At the same time, this regulatory process strengthens the plant’s antioxidant capacity, reducing oxidative damage caused by cold stress (Su et al., 2025).

In cultivated Asian rice, a distinct form of variation referred to as an epiallele has been characterized. This epiallele is marked by hypomethylation in the promoter region of the *ACT1* gene. *ACT1* loss-of-function mutants exhibit significantly reduced seed-setting rates under cold stress, whereas *ACT1*-overexpressing transgenic lines display markedly improved fertility. This study comprehensively delineates the epigenetic mechanism underlying the acquisition of heritable cold tolerance in rice, which centered on cold-induced hypomethylation of the *ACT1* promoter. Cold stress triggers transcriptional downregulation of the DNA methyltransferase MET1b, initiating promoter hypomethylation; subsequently, recruitment of the Dof1 transcription factor stabilizes *ACT1* expression under prolonged cold conditions. Critically, this environmentally responsive epigenetic variant is strongly associated with both latitudinal distribution and natural cold adaptation across diverse rice accessions, offering a robust molecular exemplar of environment-induced, transgenerationally stable epigenetic inheritance in plants (Song et al., 2025).

### 5.5 Accelerated breeding platforms

Technologies such as speed breeding are transforming the development of cold-tolerant crops through the integration of controlled environments, extended photoperiods, and precise temperature regulation, which substantially shorten breeding cycles. In wheat, the conventional process of developing homozygous pure lines is typically lengthy, often necessitating up to eight generations of self-pollination to attain genetic stability. Rapid generation advancement (RGA) protocols, however, grounded in a thorough understanding of plant physiology and photoperiod sensitivity, can markedly accelerate this process. By cultivating plants under continuous light and optimal temperatures, RGA enables the production of six to eight wheat generations per year, while also circumventing vernalization requirements in certain genotypes. This methodology can be effectively combined with modern breeding approaches, such as marker-assisted backcross breeding (MABB) and genomic selection (GS), to facilitate the concurrent selection of multiple target alleles and the improvement of complex traits within a condensed timeframe (Prashanth et al., 2022). The intelligent breeding platform facilitates the integrated application of technologies including genome editing, phenotypic analysis, and environmental monitoring. This promotes the shift of breeding processes toward automation and intelligence, while substantially reducing the development cycle for new germplasms. Herein, we consolidate the aforementioned information into **Table 1**.

**Table 1:** Summary of candidate genes: essential information and biological functions.

Gene name	Species	Technical type	Main functions or research content
COLD1	Rice	traditional overexpression	Cold receptor
COOL1	Zea mays	CRISPR/Cas9	Overexpression negatively affects cold tolerance, a crucial natural variation for high-latitude adaptation
ZmPgb1. 1	Zea mays	traditional overexpression	Regulating NO metabolism enhances cold resistance
ZmPIF6	Zea mays/Rice	heterologous overexpression	Overexpression in rice enhances cold tolerance and increases grain size
OsPOX1	Rice	RNAi	Clear ROS
OsNCED4/5	Rice	traditional overexpression	Regulate ROS homeostasis
STP2	tomato	traditional overexpression	Alleviate fruit deformities in low temperatures
MdS6PDH	apple	traditional overexpression	Cold resistance
MdDAM1	apple	RNAi	Winter Dormancy Regulation
VaSUS2	Tomato	heterologous overexpression	Increase cold resistance
CBF1	Tomat	traditional overexpression	classic CBF pathway
CBF	Arabidopsis thaliana	CRISPR/Cas9	Sensitive to low-temperature stress
ICE1	Arabidopsis thaliana	traditional overexpression	Proper tuning CBF
ZmICE1	Zea mays	CRISPR/Cas9	Sensitive to low-temperature stress
ICE41	Arabidopsis thaliana	heterologous overexpression	Increase cold tolerance
OsPIN5b	Rice	traditional overexpression	Plant height increases but cold tolerance decreases
HOS1	Arabidopsis thaliana	T-DNA insertion	Degrade ICE1
SIZ1	Arabidopsis thaliana	traditional overexpression	Stabilize ICE1
ESK1	Arabidopsis thaliana	EMS	Proline accumulation, constitutive cold tolerance
NTL8	Arabidopsis thaliana	T-DNA insertion	Vernalization Pathway
CRY2	Arabidopsis thaliana	T-DNA insertion	Cold domestication under blue light
ScF3'H	Potato	CRISPR/Cas9	Increase cold tolerance
ZmALS1/2	Zea mays	Prime Editing	Herbicide-resistant
CWINs promoter	Rice/Tomato	Prime Editing	Enhanced carbon allocation and heat tolerance

Genome-wide association studies (GWAS) in maize have been used to elucidate the genetic architecture underlying cold tolerance. A key finding is the identification of COOL1, a quantitative trait locus (QTL) linked to cold tolerance at the seedling stage. The cold-tolerant allele at this locus exhibits strong correlations with enhanced germination rates, improved seedling vigor, and broader adaptation to high-latitude environments, which are characterized by shorter growing seasons and lower temperatures early in the season. This well-defined genetic marker offers potential for use in MAS programs, facilitating the efficient introgression of the favorable allele into elite maize germplasm and accelerating the development of varieties better suited to cooler climates (Zeng et al., 2025).

These advances collectively underscore the increasing synergy between fundamental physiological insights and practical breeding efficiency. For example, knowledge of photoperiodic compensation mechanisms, where prolonged daylight exposure can partially replace the vernalization requirement for flowering in certain cereal crops, which is now being directly integrated into RGA systems. By combining such physiological manipulation with high-throughput genotyping and phenotyping platforms, a more predictive and targeted crop improvement strategy is enabled, accelerating the development of climate-resilient cultivars.

## 6 Combined stress and cross-adaptation

Plants often face multiple concurrent stresses, and their interactions exacerbate the damage. In the combined cold-drought stress, low temperature reduces the water conductivity of roots, intensifies drought, leads to high osmotic pressure, and reduces photosynthetic efficiency, far exceeding that of single stress (Kim et al., 2014). Under cold-salt combined stress, the damage to plants is more severe than that under single stress. The SOS pathway is the core mechanism for plants to respond to salt stress and maintain ion homeostasis. The ICE1-CBF cascade is the key pathway for cold signal transduction. Under combined stress, the two pathways may have cross-talk through common nodes such as the MAPK cascade, but the specific interaction mechanism needs further in-depth study (Huang et al., 2011). Under combined cold and disease stress, there is a complex interaction between plant immunity and cold signals. The latest research shows that ICE1, as a key node, integrates low-temperature and salicylic acid signals. Its interaction with NPR1 activates the expression of PR genes and positively regulates the immune response (Li et al., 2024c). However, cold signals may also negatively regulate disease resistance: After the knockout of the *SlCBF1* gene in tomatoes, the resistance of fruits to *Botrytis cinerea* is enhanced, the JA content increases while the SA content decreases, indicating that CBF1 may negatively regulate disease resistance after cold storage by regulating the SA/JA balance (Xiang et al., 2025a). Exogenous application of salicylic acid to rice under cold stress conditions significantly upregulates the relative expression levels of key genes involved in cold signal transduction (*OsCOLD1*, *OsCDPK7*, *OsMYB*), osmotic regulation (*OsCOIN*, *OsLti6a*, *OsLti6b*, *OsICE1*), and oxidative stress response (*OsTrx23*, *OsFER1*, *OsSODB*, *OsTERF2*). This treatment further enhances seed germination rate and germination index, promotes nutrient uptake in rice seedlings, increases the content of osmotic regulators such as proline and soluble proteins, and elevates cellular water potential under cold stress conditions. Furthermore, SA application reduces reactive oxygen species accumulation and mitigates peroxidative damage to the plasma membrane by enhancing the activities of peroxidase (POD), CAT, APX, and increasing ascorbic acid content. Collectively, these responses significantly enhance the cold tolerance of rice plants (Xu et al., 2025). In production practice, cold stress during the booting stage of rice often exacerbates the occurrence of rice blast. The relationship between cold acclimation of wheat and resistance to snow mold varies among varieties, and post-harvest cold stress of tomatoes promotes the infection of gray mold. These phenomena reflect the complex synergistic and antagonistic relationships between cold signals and immune pathways.

The cross-adaptation mechanism involves signal convergence (the synergistic action of H_2_O_2_, calcium signals, and MAPK cascades), the accumulation of metabolites, and epigenetic modifications. Currently, some of these mechanisms have been implemented in breeding. Drought pre-treatment can improve the cold tolerance of wheat through ABA accumulation; cold acclimation-induced adaptation can enhance the salt tolerance of Arabidopsis; heat shock treatment produces cross-protection through the accumulation of heat shock proteins such as HSP70/90, which helps to alleviate subsequent cold damage. Breeding strategies can include aggregating key genes for different stresses, targeting multi-stress regulatory nodes such as ICE1 and MAPK, constructing synthetic promoters for multi-stress responses, and establishing a multi-stress phenotypic screening platform, with yield stability as the core selection index.

## 7 Discussion and prospect

Future breeding programs for cold tolerance should prioritize precision genome editing of validated cold-responsive candidate genes, such as *OsNAC050* and *OsMYB30*, which is to maximize on-target efficiency and minimize off-target effects. To overcome the limitations of single-gene approaches, multiplex editing of functionally complementary polygenic modules is essential for achieving synergistic improvements in cold resilience without compromising yield or quality. Concurrently, robust, species-tailored genetic transformation systems must be established and integrated with editing platforms to accelerate the transition of edited lines from laboratory validation to field deployment and substantially shorten breeding timelines.

In parallel, AI offers transformative potential: machine learning and deep learning models can systematically interrogate multi-omics datasets (transcriptomic, genomic, epigenomic, and metabolomic) to identify novel cold-responsive genes and reconstruct regulatory networks, thereby enhancing the rigor and throughput of target discovery. Predictive AI models incorporating environmental parameters (e.g., temperature magnitude, duration, and diurnal fluctuation) should be developed to forecast phenotypic outcomes of edited genotypes under field-relevant cold stress conditions, enabling data-informed breeding decisions. Furthermore, AI-guided design of editing targets, integrated with molecular dynamics simulations, which can predict structural and functional consequences of edits “in silico”, significantly improving editing precision and experimental success rates.

Current challenges, including residual off-target activity, technical complexity of coordinated multiplex editing, and scarcity of high-quality, species-specific cold-stress omics data, which remain significant bottlenecks. These can be addressed through (i) engineering of high-fidelity Cas variants and optimized sgRNA design rules; (ii) establishment of community-driven, standardized multi-omics data repositories for non-model and understudied crops; and (iii) development of hybrid breeding frameworks that synergistically integrate precision editing, AI-driven prediction, and conventional phenotypic selection to dissect epistatic interactions among cold tolerance, yield, and quality loci, ultimately enabling their concurrent enhancement.

Despite advances in understanding the molecular basis of plant cold tolerance, significant challenges persist, both in uncovering fundamental mechanisms and in translating these insights into field applications. At the molecular level, cold tolerance is governed by polygenic networks characterized by intricate crosstalk among calcium signaling, hormone pathways (such as ABA and JA), ROS scavenging systems, and transcriptional regulatory cascades. Given this genetic complexity, single-gene manipulation approaches have generally fallen short of conferring robust and context-dependent resistance, as modifying individual components often leads to unintended pleiotropic effects or compensatory responses within the network.

Compounding this complexity, the perception and transduction of cold signals remain active areas of research, with substantial knowledge gaps yet to be addressed. The spatiotemporal dynamics of Ca^2+^; influx, critical for initiating early stress responses, and their precise synergistic interplay with downstream signaling pathways (Dodd et al., 2006), such as MAPK cascades and phospholipid signaling, are not yet fully elucidated. Furthermore, the identification and functional characterization of specific primary cold sensors in plant cells continue to be subjects of considerable debate. Whether these sensors are distinct from or share components with known thermosensors involved in high-temperature responses remains under investigation. Candidate mechanisms, including plasma membrane channel proteins and histidine kinases, are currently being explored. While, the translation of laboratory findings into agricultural practice is further complicated by a number of practical challenges. A major obstacle lies in the frequent inconsistency of traits, initially identified under stable, controlled laboratory settings, when these traits are deployed in dynamic field environments. Under real-world conditions, factors such as fluctuating diurnal and seasonal temperatures, variations in soil quality and moisture levels, diverse microbial communities, and interactions with pathogens introduce substantial complexity. For instance, while northern-adapted soybean cultivars show promising cold tolerance in controlled trials, they often fail to maintain yield advantages over southern varieties when subjected to prolonged and unpredictable cold stress in the field, where multiple stressors interact concurrently.

Additionally, the interplay between cold tolerance and responses to other abiotic stresses, such as drought, salinity, or nutrient deficiency, remains relatively underexplored. This represents a significant research gap, particularly in regions with variable climates where crops frequently experience multiple stressors, either sequentially or concurrently. Potential trade-offs, wherein enhanced performance under one stress condition compromises fitness under another, further complicate breeding strategies aimed at developing climate-resilient crops. Therefore, an integrated approach that investigates the crosstalk among signaling pathways for different abiotic stresses is essential for breeding varieties with robust and durable resilience.

Current research often fails to reflect real-world conditions: while most studies focus on single-stress treatments, plants in natural environments are generally subjected to multiple abiotic stresses simultaneously. Given the rapid and substantial changes in global climate, plants increasingly experience concurrent cold and drought stress, leading to altered morphological, physiological, and molecular responses. Both stresses adversely affect plant growth and yield by causing physical damage, physiological and biochemical disruptions, and molecular alterations. To enhance stress tolerance under such conditions, plants deploy a range of common and distinct physiological and molecular defense mechanisms. For instance, drought stress induces an increase in H_2_O_2_ levels in plant tissues. As a signaling molecule, H_2_O_2_ can, to some extent, improve a plant’s ability to tolerate cold stress (Aroca et al., 2003; Hussain et al., 2018). Understanding the synergistic or antagonistic effects of combined stress factors on cold tolerance is critical for improving the practical applicability of research, given that crops must adapt to dynamic, multi-stress environments to maintain productivity.

To tackle these multifaceted challenges, the development and integration of innovative, interdisciplinary approaches, spanning genomics, computational biology, and synthetic systems design, are essential. Advances in sequencing technology are expected to further lower the cost of genome sequencing, thereby enabling the acquisition of large-scale, high-throughput genomic data. This progress will support in-depth analysis of crop genome information, enhance the efficient identification of functional genes linked to desirable traits, and ultimately accelerate crop breeding processes. In genetic analysis, genotyping-by-sequencing (GBS) has significantly advanced research on cold tolerance, a classic complex quantitative trait controlled by multiple genes and their interactions with environmental factors. GBS has transformed this field by enabling high-throughput identification of genome-wide single nucleotide polymorphism (SNP) markers. The resulting increase in marker density substantially improves the resolution of genetic linkage maps, which in turn enhances the precision and accuracy of QTL mapping. Consequently, researchers are now better positioned to identify chromosomal regions harboring key functional genes and to elucidate the regulatory networks involved in cold stress responses, as demonstrated by foundational studies in various plant species (Bhat et al., 2016; Pan et al., 2023).

Concurrently, artificial intelligence (AI) and machine learning (ML) provide a robust framework for managing and interpreting complex biological datasets (Farooq et al., 2024). AI and big data analytics will be deeply integrated into the molecular breeding process. By consolidating multi-dimensional data such as genomic, environmental, and phenotypic information, precise breeding prediction models can be constructed to optimize the breeding decision-making system. These computational tools offer considerable potential for integrating heterogeneous multi-omics data, such as genomics, transcriptomics, proteomics, and metabolomics, alongside detailed environmental variables and high-dimensional phenotypic records. Using predictive modeling, AI can uncover nonlinear gene-gene (epistatic) and gene-environment interactions that are difficult to detect with conventional statistical methods. Under cold conditions, the NLA protein accumulates *in vivo*: on one hand, it ubiquitinates and degrades the JA signaling suppressor JAZ11, thereby activating the JA-mediated codl signal transduction pathway and enhancing cold tolerance in maize; on the other hand, NLA utilizes its SPX domain to perceive inositol polyphosphates (InsPs), leading to the recognition and ubiquitination-mediated degradation of the phosphate transporter PT4, thereby suppressing phosphate uptake in roots. These findings indicate that NLA functions as a molecular hub, simultaneously enhancing cold tolerance in maize and restricting phosphate uptake, which exemplifying a classical trade-off between traits. To overcome this trade-off, the research team integrated AlphaFold3-based structural predictions with molecular docking analyses to precisely identify the key InsPs-perception region within the SPX domain. Using CRISPR/Cas9 technology, a 12-base pair deletion was introduced into the NLA gene, resulting in the NLA^Δ12^ variant. Functional analysis revealed that the variant exhibited an approximately 50-fold reduction in InsPs binding affinity, nearly abolishing its ability to sense InsPs. Consequently, NLA^Δ12^ no longer binds to or degrades PT4, allowing phosphate uptake to be maintained. Meanwhile, the interaction between NLA and JAZ11 is independent of InsPs levels; thus, NLA^Δ12^ retains the ability to effectively degrade JAZ11, sustaining or even enhancing the JA-mediated cold tolerance pathway. This structure-informed precision editing successfully achieved functional decoupling in maize, preserving cold tolerance while relieving the constraint on phosphate uptake. This breakthrough resolves a long-standing trait trade-off at the molecular level and highlights the considerable application potential of AI-assisted protein design and precise genome editing in improving complex crop traits (Liao et al., 2026). This capability supports the development of reliable predictive models for cold tolerance, which can be leveraged to optimize breeding strategies by simulating and selecting advantageous genetic combinations to enhance resilience.

Furthermore, synthetic biology provides a complementary set of tools for the rational design and engineering of biological systems to improve cold tolerance. This approach allows the creation of novel, orthogonal genetic modules, such as engineered cold-responsive promoters or synthetic transcription factors that activate at specific cold thresholds. These synthetic circuits can be incorporated into plant systems to reprogram or fine-tune endogenous stress-response pathways, thereby enhancing cold adaptation while minimizing undesirable pleiotropic effects on normal growth and development. This methodology enables more precise control over physiological processes. Synthetic promoters are typically engineered through novel combinations of cis-regulatory elements (CREs) to modulate transgene expression (Ali and Kim, 2019). Current technological approaches involve computational selection algorithms that utilize CRE databases for the design of synthetic promoters. For instance, by integrating predictions for CREs responsive to biotic or abiotic stimuli with the 35S CaMV core promoter, several minimal core promoters have been successfully synthesized (Cai et al., 2020; Yang et al., 2021). To achieve more precise construction of synthetically regulated promoters, additional native promoter components surrounding plant core promoters have been systematically screened (Jores et al., 2021). This strategy has identified active enhancers or silencers, thereby augmenting the activity of basic core promoters through transient expression assays (Jores et al., 2020). Moreover, orthogonal regulatory systems can be devised to attain specific control and enhanced sensitivity of synthetic promoters. In one such experimental design, randomly selected yeast promoter fragments were synthesized and demonstrated to function as activators, repressors, or promoters in *Arabidopsis thaliana* and tobacco, respectively.

These advanced technologies are synergistically integrated with established modern molecular breeding techniques, including MAS, transgenesis, and CRISPR-Cas9-mediated gene editing. MAS facilitates the efficient introduction of desirable QTLs or genes into elite breeding lines. Transgenesis enables the direct incorporation of beneficial genes from distantly related species, while gene editing allows precise modifications within the native genome to enhance the function of endogenous cold-tolerance genes. The convergence of these methods supports the accurate deployment of key genetic factors, promotes targeted improvement of crop germplasm, and accelerates the development of varieties with significantly enhanced cold tolerance.

By systematically linking mechanistic insights from cold stress biology with translational agricultural innovations, current research on plant cold tolerance is well-positioned to address existing challenges. This integrated approach is critical for advancing the development of next-generation, climate-resilient crops, thereby supporting global agricultural productivity and sustainability in the face of climate change.
